# Biomarker Research in Glucose Disorders: Current Concepts and Clinical Applications

**DOI:** 10.1155/2013/290671

**Published:** 2013-06-17

**Authors:** D. R. Webb, K. Herbert, Melanie J. Davies, K. Khunti, N. Sattar, C. D. A. Stehouwer

**Affiliations:** ^1^Diabetes Research Unit, University of Leicester, Leicester General Hospital, Leicester LE5 4PW, UK; ^2^Department of Cardiovascular Sciences, University of Leicester, Clinical Sciences Wing, Glenfield General Hospital, Leicester LE3 9QP, UK; ^3^Department of Health Sciences, University of Leicester, Princess Road West, Leicester LE1 6TP, UK; ^4^BHF Glasgow Cardiovascular Centre, Wolfson Medical School Building, University Avenue, Glasgow G12 8QQ, UK; ^5^Maastricht University Medical Centre, P.O. BOX 5800, 6202 AZ Maastricht, The Netherlands

The National Institute for Health working group broadly define a “biomarker” as an objectively quantifiable characteristic reflecting normal biological action, a specific pathogenic process, or pharmacologic response to therapeutic intervention.

This meaning is readily applied to an increasing array of analytes and molecules which may enhance our understanding of disease pathogenesis, aid risk stratification for diagnosis or management, or improve treatment response.

Biomarker measurements have traditionally targeted a specific pathway or mechanism proposed to be involved in a disease, for example, association of adiponectin with the development of Type 2 diabetes or glycosylated haemoglobin in development of microvascular complications. Increasingly employed are state-of-the-art metabolomic and proteomic approaches which can reflect instantaneous cellular multiparametric responses to environmental stimuli. We now have tools to explore the genome (DNA), transcriptome (mRNA), proteome (protein), and metabolome (small molecule metabolites) for such purposes. Diabetes lends itself to all of these applications as it is clear that the pathogenesis of the disease and its complications are highly complex and involves numerous biological axes. Sensitive techniques such as nuclear magnetic resonance and gas/liquid chromatography mass spectrometry now have the capacity to process large numbers of samples and in conjunction with robust bioinformatics should enable detailed characterisation of the “metabotype.”

In this special issue we see examples of relevance to diabetes, including state-of-the-art tandem mass spectrometry measurement of L(+) and D(−) Lactate, proteomic profiling using isobaric tagging (iTRAQ) in patients with microalbuminuria, and two studies characterising the adipocytokine adiponectin within populations at risk of cardiovascular disease and diabetes. Also included is cross-sectional evidence assessing urine F2-isoprostane- and Intercellular Adhesion Molecule-1 (ICAM-1) as biomarkers of oxidative stress and endothelial function, respectively.

Biomarker research to date has generally disappointed by failing to provide additional predictive power over and above existing classical risk factors or only minimally advancing our understanding of diabetes. Hopefully utilising existing biomarker knowledge in a targeted fashion in addition to exploiting novel “–omics” technologies will generate more useful biomarkers which are independent of known risk factors and shed new light on disease pathogenesis.

